# Nondestructive Detection of Weight Loss Rate, Surface Color, Vitamin C Content, and Firmness in Mini-Chinese Cabbage with Nanopackaging by Fourier Transform-Near Infrared Spectroscopy

**DOI:** 10.3390/foods10102309

**Published:** 2021-09-29

**Authors:** Qiang Liu, Shaoxia Chen, Dandan Zhou, Chao Ding, Jiahong Wang, Hongsheng Zhou, Kang Tu, Leiqing Pan, Pengxia Li

**Affiliations:** 1 College of Food Science and Technology, Nanjing Agricultural University, Nanjing 210095, China; qiangliu@nufe.edu.cn (Q.L.); 2017108049@njau.edu.cn (S.C.); kangtu@njau.edu.cn (K.T.); 2 College of Food Science and Engineering, Nanjing University of Finance and Economics, Nanjing 210023, China; cding@nufe.edu.cn; 3 College of Light Industry and Food Engineering, Nanjing Forestry University, Nanjing 210037, China; dandanz@njfu.edu.cn (D.Z.); njfuwjh@njfu.edu.cn (J.W.); 4 Institute of Agricultural Facilities and Equipment, Jiangsu Academy of Agricultural Sciences, Nanjing 210014, China; hongshengzhou@jaas.ac.cn

**Keywords:** near infrared spectroscopy, nondestructive detection, mini-Chinese cabbage, nanopacking, storage

## Abstract

A nondestructive optical method is described for the quality assessment of mini-Chinese cabbage with nanopackaging during its storage, using Fourier transform-near infrared (FT-NIR) spectroscopy. The sample quality attributes measured included weight loss rate, surface color index, vitamin C content, and firmness. The level of freshness of the mini-Chinese cabbage during storage was divided into three categories. Partial least squares regression (PLSR) and the least squares support vector machine were applied to spectral datasets in order to develop prediction models for each quality attribute. For a comparative analysis of performance, the five preprocessing methods applied were standard normal variable (SNV), first derivative (lst), second derivative (2nd), multiplicative scattering correction (MSC), and auto scale. The SNV-PLSR model exhibited the best prediction performance for weight loss rate (*R_p_*^2^ = 0.96, RMSEP = 1.432%). The 1st-PLSR model showed the best prediction performance for L* value (*R_p_*^2^ = 0.89, RMSEP = 3.25 mg/100 g), but also the lowest accuracy for firmness (*R_p_*^2^ = 0.60, RMSEP = 2.453). The best classification model was able to predict freshness levels with 88.8% accuracy in mini-Chinese cabbage by supported vector classification (SVC). This study illustrates that the spectral profile obtained by FT-NIR spectroscopy could potentially be implemented for integral assessments of the internal and external quality attributes of mini-Chinese cabbage with nanopacking during storage.

## 1. Introduction

Mini-Chinese cabbage (*Brassica campestris*) originated in China, and is one of the most frequently consumed Brassica vegetables available in the market owing to its fresh appearance, high nutrient content, special texture, and the presence of vitamins and other valuable metabolites [1]. In general, as a cool-season leafy vegetable, mini-Chinese cabbage is grown in greenhouses. However, unlike other root vegetables and fruits such as tomato, carrot, and onion, mini-Chinese cabbage is typically harvested when it gets ripened with high moisture content, i.e., in the range between 90 and 95%. Nonetheless, it is highly susceptible to weight loss or spoilage, and thus is prone to quality deterioration during postharvest storage. Therefore, changes in quality of mini-Chinese cabbage during storage have been extensively investigated by several researchers [2,3]. The techniques used so far for quality assessments have included chemical analysis, preservation, and biological activity. Monitoring the quality attributes of mini-Chinese cabbage during storage and transport can be challenging for the vegetable industry. Currently, simple visual inspection and touch-based chemical/physical examination are the two most widely-used methods for determining the quality of fresh products. However, such methods have several weaknesses, e.g., they cannot be applied on a large scale. Although chemical analysis can provide details with high accuracy, it is usually time-consuming, requiring tedious sample preparation procedures, and cannot simultaneously evaluate the internal and external attributes [4,5,6]. For these reasons, the development of fast, sensitive, and nondestructive approaches to detect the quality attributes is of significant commercial interest.

It is noteworthy that vegetables without packing procedures are particularly prone to quality deterioration. Nanopacking, a packing procedure, can dynamically control environmental factors including oxygen and moisture concentration during storage [7]. Recently, a concept involving the application of nanocomposites was introduced into the food packaging industry, with the objective of extending loquat storage life [8], inhibiting browning and maintaining the quality of fresh-cut sugarcane [9], and maintaining the quality of green asparagus in storage [10]. Notably, the accumulation of reactive oxygen species (ROS) and phenolic compounds can play an active role in preventing cells in vegetables from suffering oxidative damage during storage [11]. Chemical components including soluble sugar and ascorbic acid act not only as nutrients, but also as antioxidants in vegetables [8]. However, the storage period of nanopackaging is quite complex, and the metabolic activity of vegetables can result in significant changes both chemically and physically, including enzyme activity, respiratory rate, weight loss, and surface color changes. The changes of nutritional composition which occur after nanopackaging are quite different from those that take place under the normal storage. Thus, it would be very interesting to monitor the quality attributes in food with nanopackaging during storage.

Nondestructive detection techniques for the determination of the internal and external quality attributes of vegetables have been widely studied in the food industry. The most commonly employed wavelength range in the near infrared (NIR) region is 780 to 2526 nm. The chemical bands originating from *X–H* (e.g., *C–H*, *O–H*, *N–H*) vibrations are strongly articulated in the NIR region. Moscetti et al. [12] devoted extensive research efforts and successfully developed NIR spectroscopy with chemometrics for the prediction of water activity (R^2^ = 0.91) and water content (R^2^ = 0.97) in organic carrot slices. Botros et al. [13] used NIR spectroscopy with chemometrics to successfully identify authentic cow milk powder. Literature studies [14,15] have also presented comparative analyses of the spectroscopy with various wavelength ranges for the prediction of chemical attributes in food composition. Giovenzana et al. [16] presented an overview of NIR spectroscopy applications for the assessment of vegetables, focusing on different aspects of the distribution process. Our previous study also demonstrated that imaging information in the NIR wavelength range was useful for predicting soluble sugar content in strawberries, with *R_p_*^2^ of 0.807 [17]. While most recent studies using NIR spectroscopy have focused on intact fruit, evaluations of the freshness of vegetables during storage, especially when novel storage approaches (i.e., nanopackaging) are used, have not been widely reported. To the best of our knowledge, to date, the application of NIR spectroscopy for the nondestructive detection of quality attributes in mini-Chinese cabbage has never been investigated, and only minor research efforts have been focused on the motoring of the inspection of quality attributes in vegetables with nanopackaging. Given the advantages of NIR spectroscopy for the nondestructive evaluation of fresh agri-products, studies should better extend the detection application to include leafy vegetables and novel storage approaches.

The main objectives of this study are as follows: (1) to acquire the NIR spectral profiles for mini-Chinese cabbage with nanopackaging during storage; (2) to analyze the correlations between spectral profiles and the corresponding quality attributes in mini-Chinese cabbage during nanopackaging; (3) to develop prediction models based on NIR spectroscopy with chemometrics; and (4) to validate optimized pretreatments in the NIR dataset, providing the best results in predictions of freshness grade of mini-Chinese cabbage under nanopacking.

## 2. Materials and Methods

### 2.1. Samples and Preparation

Mini-Chinese cabbages (Brassica campestris. *Var. Xialing*), harvested after a 50 d growth period, were purchased from a local vegetable market in Nanjing, China (32°07′35″ N and 118°59′27″ E). Samples with uniform ripeness, shape, and surface color were selected. Then, the cabbages were immediately transported to the College of Food Science and Technology at Nanjing Agricultural University for precooling treatment (4 °C for 24 h). All 288 samples were washed with distilled water, and water from the surface was naturally evaporated at room temperature (25 °C) for 4 h. All samples were packed using nanopackaging bags and stored at a temperature of 20 ± 0.5 °C and relative humidity of 85 ± 5%. Polyethylene (PE) packaging bags (without nanopacking material) were used as a second group, and nonpackaged cabbages were used as the control. The total duration of the storage period was 15 days: (1) Six samples for nanopacking, PE-packing, and control (nonpacking) conditions (a total of 108 samples) were randomly selected every 3 d, and were used for physiochemical analysis; (2) another 180 samples with different freshness levels were selected to obtain the NIR spectral profiles, followed by chemometrics for analysis and the modeling of quality attributes.

### 2.2. Nanopacking Material

In this study, the method proposed by Li et al. [18] was used to prepare the nanopackaging material. Briefly, nanopower (30%, nano-Ag:nano-TiO_2_:nano attapulgite:nano-SiO_2_ = 6:7:5:2), linear low density PE (68%), and coupling reagents (2%) were uniformly blended using a high-speed mixer at a speed of 600 rpm for 1 h (Type: SK-1, Ronghua Instrument Co. Ltd., Changzhou, China). After cooling for 1–2 min, the mixture was cut into nanogranules. According to the mass ratio, 3.75% nanogranules, 3.75% antifogging agents, and 92.5% PE granules were mixed and stirred for 30 min, and prepared in a film with a thickness of 40 μm. Finally, all nanopackaging bags were cut with size of 40 cm · 15 cm size for use in the described storage method. The mechanical properties of the nanopacking material were tested using an electronic universal testing machine (Type: 3369, Instron Corp., Boston, MA, USA). The tensile strength of the nanopacking material was 20 MPa and the tensile elongation was 22.0%. The CO_2_ transmission rate for the nanopacking bags was measured according to the National standards of China (GB/T 1038-2000 Plastics film and sheeting determination of gas transmission differential pressure method), and determined to be 0.102 cm^3^/(m^2^·h·Pa).

### 2.3. FT-NIR Spectroscopy

The spectral profiles of mini-Chinese cabbage were acquired by FT-NIR spectroscopy (Antaris II, Thermo Scientific Inc., Waltham, MA, USA). The system was equipped with a light source, a beam splitter, a InGaAs detector, and a data processing unit. A handheld fiber optic sampling probe (Scientific SabIR Fiber Optic Probe, Thermo Scientific Inc., Waltham, MA, USA) with a bifurcated fiber bundle to irradiated the sample and collect the reflected/scattered light was used. NIR spectroscopy data were collected under diffuse reflection mode; the scanning range was set from 4000 to 10,000 cm^−1^ and the scanning resolution was 4 cm^−1^. To obtain a stable signal, the average spectrum after 32 scans was recorded as the sample spectrum.

Based on a study by Ibáñez et al. [19], measurements of NIR spectra were carried out at three different, equidistant points with 120° angle intervals for each sample. Previous works have demonstrated that FT-NIR can be used successfully to evaluate the quality attributes of vegetables [20,21]. While this work focuses on the quality attributes of mini-Chinese cabbage with nanopacking, and regression models for quality attribute determination in nanopacking using FT-NIR were developed, the spectral datasets in the control and PE-packing groups were not taken into consideration.

### 2.4. Quantitative Analysis of Surface Color, Weight Loss Rate, Vitamin C and Firmness

Color measurement in stored samples was determined using a Minolta Chroma Meter (type: CR-13, Konica Minolta Inc., Tokyo, Japan), and index values of L*, a*, and b* were obtained. Total color difference Δ*E* was calculated as following:

$$\Delta E=\sqrt{{\left(L^{*}-{L_{0}}^{*}\right)}^{2}+{\left(a^{*}-{a_{0}}^{*}\right)}^{2}+{\left(b^{*}-{b_{0}}^{*}\right)}^{2}}$$

where *L*_0_*, *a*_0_* and *b*_0_* are the original color indexes of the sample. Analyses were performed in triplicate, and mean values of L*, a*, b* and Δ*E* were calculated for data analysis.

Weight loss rate was determined using the following equation:

$$WR=\frac{OW-WS}{OW}\times 100\%$$

where *WR* indicates weight loss rate, *OW* is the original weight of the sample, and *WS* is the weight of the sample after storage. Experiments were carried out in triplicate on each sample, and the mean value of weight loss rate was calculated for analysis.

Firmness was measured using a texture property analyzer (type: TA.XT Plus, Stable Micro System Inc., Surrey, British) [21]. A P6 cylindrical probe with a 6-mm dimeter was selected. The preparation rate of the probe was 5.00 mm/s and the measurement rate was 1.00 m/s. Penetration mode was used in this experiment, and the measured height was 6.00 mm. The maximum force (unit: N) in the pressing process was applied as the firmness value of the mini-Chinese cabbage. Experiments on each sample were carried out in triplicate, and the mean value of firmness was calculated for analysis.

Vitamin C (Vc) content was measured by the 2,6-dichloroindophenol titrimetric method [22] with some modifications. Mini-Chinese cabbage (5 g) was added to a metaphosphoric acid–acetic acid solution (50 mL, 5%). Then, the mixed solution was filtered and 10 mL of filtrate was titrated with 2,6-dichloroindophenol standard solution. Standard solutions with known concentration (0, 5, 10, 20, 50, 100 mg/100 g) of ascorbic acid (analytical pure) in ultrapure water were used to obtain the standard curves. Experiments on each sample were carried out in triplicate and the mean value of Vc was calculated for data analysis; values were expressed as mg/100 g.

### 2.5. Freshness Levels Description

Applying the Chinese official analysis method (NY/T 943-2006: Grades and specifications of Chinese cabbage) with some modifications, the freshness levels of the cabbage were visually inspected and determined based on weight loss rate, L* value, and Vc content. The quality characteristics of cabbages during storage and the related specifications are presented in Table 1.

### 2.6. Data Processing

The methodology used in our previous study [17] was applied with some modifications for the present data analysis. Five preprocessing methods, namely, auto-scale, standard normal variable (SNV), multiplicative scattering correction (MSC), first derivative (1st), and second derivative (2nd), were utilized to compensate for baseline shifting, noise removal, uniform light scattering, etc. These methodologies were recommended for practical applications in [23].

In this study, the linear multivariate algorithm (partial least square regression, PLSR) and nonlinear multivariate algorithm (support vector regression, SVR) were used to build the regression models for predicting the quality attributes of the samples. Classification models for freshness level were developed using partial least square-discrimination analysis (PLS-DA) and supported vector classification (SVC). The dataset with 180 samples was divided in a ratio of 3:1 using the Kennard Stone algorithm [24]. Three quarters of the cabbages comprised the calibrated datasets, while remaining quarter was used in prediction optimization.

### 2.7. Evaluation of Models

Key parameters, namely coefficient of determination for calibration (*R_c_*^2^), coefficient of determination for cross-validation (*R_cv_*^2^), coefficient of determination for prediction (*R_p_*^2^), root mean square error of calibration (RMSEC), root mean square error of cross-validation (RMSEP), and root mean square error of prediction (RMSEP) were considered. Additionally, the practical utility of the calibration models was further assessed using the ratio of prediction to deviation (RPD). Good prediction models exhibit high values of R^2^ and RPD but low values of RMSE [25]. The parameters were defined as follows:

$${R_{c}}^{2},{R_{cv}}^{2},{R_{p}}^{2}=1-\frac{\sum_{i=1}^{N}{\left(y_{i}-\bar{y}\right)}^{2}}{\sum_{i=1}^{N}{\left(y_{i}-y_{m}\right)}^{2}}$$

$$\mathrm{RMSEC},\mathrm{RMSECV},\mathrm{RMSEP}=\sqrt{\frac{1}{N}\sum_{I=1}^{N}{\left(y_{i}-\bar{y}\right)}^{2}}$$

where *N* is the number of samples, *y_i_* and $\bar{y}$ are the measured and predicted values of the sample, and *y_m_* is the average value of all samples in the calibrated and predicted sets.

All FT-NIR data were collected using Thermo Scientific RESULT software. Multivariate statistical analysis with different preprocessing and regression methods were constructed and compared, respectively, using PLS_toolbox 7.5 (Eigenvector Reseatrch Inc., Manson, WA, USA) in Matlab (version: 2014a, the MathWork Inc., Natick, MA, USA). To avoid overfitting, validation samples were not used in the calibration and cross-validation steps [15]. The leave-one-out methodology, which does not waste data and is suitable for small numbers of samples, was utilized for optimal parameter selection.

### 2.8. Statistical Analysis

One way analysis of variance (ANOVA) was used to test the significant difference of mean values among all groups during storage. Principal component analysis (PCA) was performed using the SPSS software (version: 18.0, IBM Co., Armonk, NY, USA). Data were expressed as average value ± standard deviation (SD). *p* < 0.05 was considered to represent a significant difference. All figures were depicted using the Origin software (version: 9.0, Origin Lab Co., Northampton, MA, USA).

## 3. Results and Discussion

### 3.1. Analysis of Surface Color, Weight Loss Rate, Vitamin C and Firmness for Mini-Chinese Cabbage during Storage

**Figure 1:**
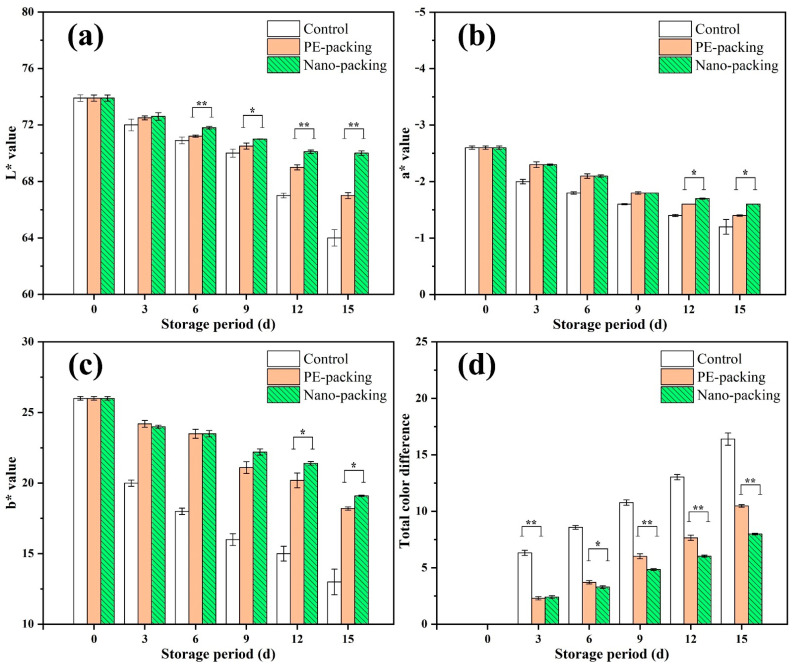
Bar charts of L* (**a**), a* (**b**), b* (**c**) and total color difference (**d**) of mini-Chinese cabbage during storage from 0 to 15 d. * and ** indicate significant difference at *p* < 0.05 and *p* < 0.01, respectively. Data are expressed as mean ± standard error.

The surface color indexes, i.e., L*, a*, and b*, are expressed in Figure 1. The L* and b* values of the exterior surface of the samples gradually became lower with storage period (Figure 1a,c), while a* showed a slight increase during storage (Figure 1b). Samples without packing (control group) showed a more obvious decreasing/increasing trend in the aforementioned curves than those subject to nanopacking and PE packing, i.e., the initial L* and b* values of 73.9 and 26.0 decreased to 64.0 and 13.0, respectively. However, limited declines were observed in the packing groups: L* was 67.0 and 70.0, and b* was 18.2 and 19.1 for PE-packing and nanopacking, respectively. Comparatively, L* showed the most sensitivity in early storage. After 6 d storage, L* was significantly different (*p* < 0.01) between the nanopacking and PE-packing samples, while nonsignificant differences were observed for a* and b*. Figure 1d reports the total color changes (Δ*E*) of mini-Chinese cabbage during storage from 0 to 15 d. An increasing trend among all groups during storage is evident. A significant difference was observed between the control and the packed samples at 3 d. After storage for 6 d, it was observed that the Δ*E* in the PE-packing group was significantly different for that of the nanopacking group. This result suggested nanopacking can effectively prolong the stability of the surface color of mini-Chinese cabbage during storage.

**Figure 2:**
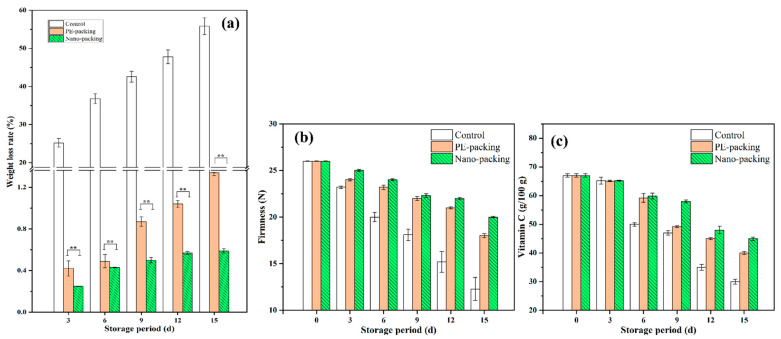
Bar charts of weight loss rate (**a**), firmness (**b**) and Vc (**c**) of mini-Chinese cabbage during storage from 0 to 15 d. ** indicate significant difference at *p* < 0.05 and *p* < 0.01, respectively. Data are expressed as mean ± standard error.

The changes of weight loss rate, firmness, and Vc are shown in Figure 2. The average weight loss rate of the samples was very predictable for the two packing groups; a ratio of 0.59 % was observed after 15 d storage in nanopacking, while that value was 1.34 % for the PE-packing group. Similar results were observed for firmness and Vc content; see Figure 2b,c. The firmness decreased in the nanopacking group (indicating that the texture of the cabbage had been well preserved) from 26.0 N to 20.0 N after 15 d storage. The average Vc contents decreased to 30.8 g/100 g, 40.0 g/100 g, and 45.0 g/100 g for control, PE-packing, and nanopacking samples, respectively, at the end of the storage period. The data showed that the packing type was significantly correlated with surface color, weight loss rate, firmness, and Vc, suggesting that the packing method is an important determinant of mini-Chinese cabbage preservation. During storage, cell water loss causes plasmolysis, which affects cell size and leads to decreased cell adhesion. Subsequently, intercellular spaces appear, and cell walls collapse, which can result in a decrease in firmness [26].

Mini-Chinese cabbage is highly perishable due to its high moisture content and the enzyme activities in fresh leaves after harvest, making it particularly susceptible to browning and the loss of nutrients during storage. Packing materials are play a key role in the storage of vegetables. In particular, nanopacking (with nano-Ag, nano-TiO_2_, and nano-SiO_2_) has been shown inhibit weight loss and color browning in mini-Chinese cabbage during storage. Similar results were also reported by Wang et al. [27], suggesting that packing bags containing nano-SiO_2_ can extend the shelf life of rice. Compared with PE packing, a greater accumulation of ROS induces damage to oxidative and membrane lipids, thereby preventing browning (lower L* value) and loss of Vc [28]. Low oxygen permeability can be achieved with nano-SiO_2_ packing materials, which can reduce enzyme activities and can prevent the oxidation of Vc [19]. Evaluating these chemical and physical changes is critical for determining the performance of nanopacking materials for use with fresh vegetables. Thus, the following quantitative predictions for physicochemical attributes after nanopacking treatment were made.

### 3.2. Spectral Analysis

**Figure 3:**
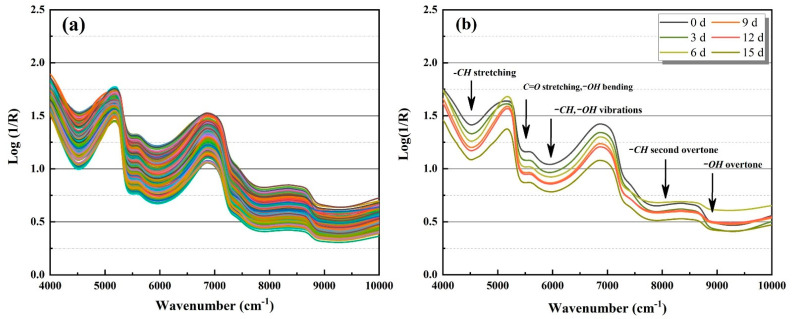
Relative reflectance spectra of mini-Chinese cabbage in nanopacking obtained using FT-NIR from 4000 to 10,000 cm^−1^. ((**a**) Individual sample; and (**b**) mean spectra during each storage period).

As stated in Lambert-Beer law, using the ratio of the outgoing intensity of a light beam to the incoming intensity transmittance, the measured transmittance concentration can be converted to absorbance and expressed as $Log\frac{1}{R}$ [29]. The values of relative reflectance spectra for individual mini-Chinese cabbage samples with nanopacking ranged from 4000 to 10,000 cm^−1^, as plotted in Figure 3a. Mean spectral curves in 0, 3, 6, 9, 12 and 15 d were calculated and are plotted in Figure 3b. The spectral profiles of the cabbages after different storage periods were found to be similar. The main absorption peaks were located at around 4600, 5170, 5900, 7900, and 8990 cm^−1^. NIR spectra remain the most comprehensive source of information about the aharmonicity of molecular vibrations [30]. These absorption wavenumbers are related to –*CH*, *C=O*, and –*OH* chemical bonds, which are the primary structural components of organic molecules [31]. A peak at around 4600 cm^−1^ is related to the –*CH* stretching [32]. The absorption peak found near 5170 cm^−1^ is mainly attributed to the *C=O* stretching and –*OH* bending. Notably, the prominence of the first overtone band of C=O stretching mode (5260–5130 cm^−1^) varies strongly among different molecular systems [30,33]. The combination band of –*CH* and –*OH* vibrations is positioned near 5900 cm^−1^. The spectrum of the cabbage displayed the second overtone of –*CH* at around 7900 cm^−1^. Finally, a strong absorption peak located at 8990 cm^−1^ was attributed to the second overtone of *O–H* stretching [34].

Figure 3b demonstrates that the spectral absorption of the samples with nanopackaging gradually weakened during 15 d storage at 20 °C. The results indicated that the difference in spectral profiles were possibly related to variations in the physiochemical properties in the samples during storage. As mentioned above, nanopacking can be utilized retain freshness in mini-Chinese cabbage. The abundance of hydrogen-containing chemical bands (i.e., high Vc and free water content) in samples forms the theoretical basis for obtaining information about the chemical structure in NIR analyses. In this study, the broad absorption peak at around 4600 cm^−1^ was closely related with –*CH* chemical bonds in Vc. The peaks at around 5170 and 8990 cm^−1^ were mainly due to the fact that the packaging treatment can inhibit the breathing and transpiration of the baby cabbage and reduce loss of water [35,36]. Further analyses were carried out using multivariate algorithms to evaluate the nondestructive and fast prediction models, which were subsequently tested.

**Figure 4:**
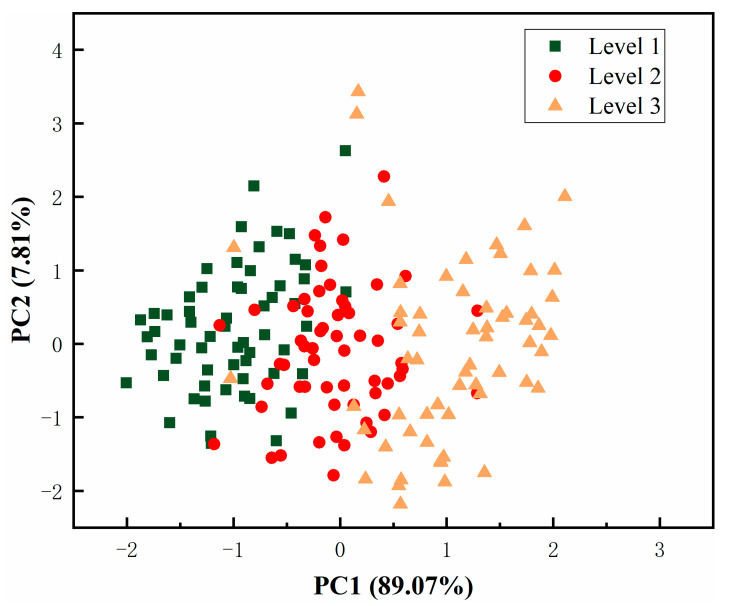
Loading plot of PC1 and PC2 derived from raw spectra for mini-Chinese cabbage at different freshness levels.

Figure 4 shows the score plot of PC1 and PC2 derived from raw spectra for mini-Chinese cabbage at different freshness levels. PC1 accounted for the highest ratio, i.e., 89.07% in the raw data, while the PC2 accounted for 7.81%. The accumulative contribution ratio of PC1 and PC2 was higher than 95%, indicating that the features in these two were aggregative [37]. Since there were slight overlaps in the different freshness levels, an obvious separation trend could be observed in the samples. Similar result were observed in studies of the shelf-life of strawberries using visible and near infrared spectroscopy [38]. Raw spectral data obtained using FT-NIR usually present excellent resolution, and the acquired matrix often contains hundreds of wavelengths. Dimension reduction is essential to improve data analyses in terms of the number of calculations and robustness. In this work, a loading plot of the PCA results can be used to reveal the relationships among the FT-NIR spectra and quality attributes.

### 3.3. Prediction Performance of Surface Color and Quality Attributes Based on FT-NIR Dataset

In this section, details are presented of the multivariate chemometrics analysis used to predict the surface color and quality attributes of mini-Chinese cabbage via FT-NIR spectroscopy. The performance of linear and nonlinear regression models combined with five different pretreatment methods (namely SNV, 1st, 2nd, MSC, and Auto scale) was compared, and is summarized in Table 2 and Table 3. Prediction models using PLSR exhibited the best weight loss rate prediction, with the *R_p_*^2^ values varying from 0.80 to 0.96, RMSEP from 1.332 to 1.564%, and RPD from 2.191 to 3.612, while the worst prediction accuracy was obtained from SVR models. Among the five pretreatment methods, the best predicted model based on the full wavebands of FT-NIR datasets achieved 0.96 of *R_p_*^2^ and 3.612 of RPD by SNV-PLSR.

For firmness predictions, the PLSR models showed poor performance, with *R_p_*^2^ values varying from 0.40 to 0.58, RMSEP from 2.578 to 2.695 N, and RPD from 1.128 to 2.342. Similar results for firmness predictions were observed for SVR models. The best result was obtained using Autoscale-SVR, with 0.60 of *R_p_*^2^ and 2.205 of RPD, which indicated that the PLSR and SVR models are not suitable for nondestructive predictions of firmness in mini-Chinese cabbage. This may be attributable to the limited range of the changes of firmness in mini-Chinese cabbage subject to nanopacking.

Furthermore, to predict the Vc content, high performance was achieved, with *R_p_*^2^ varying from 0.78 to 0.95 and RPD from 2.015 to 2.883 by PLSR, and *R_p_*^2^ from 0.79 to 0.87 and RPD from 2.013 to 2.738 by SVR. The MSC-PLSR model exhibited the best performance among all of the constructed models for Vc predictions. These results are consistent with those of Liu et al. [39]. The predictive capacity of the surface color indexes (L*, a*, and b*) is presented in Table 2. The best predicted *R_p_*^2^, RMSEP, and RPD for L* were, respectively, 0.82, 2.013, and 3.069 using MSC-SVR; for a*, the values were 0.73, 1.288, and 2.145, obtained using SNV-SVR; and b* 0.85, 1.264, and 2.432 using 1st-PLSR. These results are slightly better than those obtained by Balage et al. [40] in predictions of the attributes of pork samples, and similar to those reported by Li et al. [41] for assessments of the color indexes in plums subject to low-temperature storage.

According to the literature [39], an RPD should be greater than 2.0 for reasonable predictions, while a value of over 3.0 indicates reliable predictions. Thus, the models proposed in this study for weight loss rate and L* can be utilized for analytical purposes, and the prediction models for all attributes can be considered to be reliable. Moreover, SVM performed consistently better than PLS in predicting total soluble solids, Vc content, and titratable acid [39], which indicates that SVM is more capable of handling noisy and high-dimensional data. However, in this study, there was inefficient evidence to determine whether PLSR performed better or worse than SVR for quality attributes in mini-Chinese cabbage with nanopacking. An analysis and comprehensive comparison of the pretreatment methods indicated that SNV, MSC, and Auto scale performed best in terms of improving the model performance for weight loss ratio, Vc content, and firmness. The second model did not improve the prediction accuracy for all attributes, which is in good agreement with the results reported by Balage et al. [40]. In the present study, nanopacking positively correlated with reduced deterioration in the quality of mini-Chinese cabbage during storage. The speed of enzyme reaction and the breakdown of cell wall in the leaves were reduced, and thus, the surface color and tissue structure were preserved. Fresh cabbage contains more free water, and the physiological status of the microstructure results in stronger light scattering [41]. Thus, more light gets scattered (or reflected) backwards which can then be captured by the NIR detector.

### 3.4. Classification Performance of Freshness Levels Based on FT-NIR Dataset

According to the descriptions of freshness levels provided above for mini-Chinese cabbage subject to nanopacking storage, the classification models for different freshness levels were constructed based on full wavelength FT-NIR datasets. FT-NIR datasets comprised 180 samples, and were defined as independent variable *X*, while freshness level (measured using the reference methods presented in Section 2.6) was defined as classified or dependent variable *Y*. The classification results are presented in Table 4 and Table 5. In PLS-DA, accuracies of 82.1% for calibration and 79.9% for prediction were observed. The models obtained significantly higher accuracies, i.e., 89.6% for calibration and 88.8% for prediction, when SVC was applied. These demonstrations showed that FT-NIR spectroscopy could be successfully applied to classify the freshness level of mini-Chinese cabbage subject to nanopacking storage. Furthermore, the results indicated that the classification models based on SVC methods offer substantial potential to predict the freshness level of mini-Chinese cabbage. The model could be utilized for the further design of an online system for quality monitoring when nanopacking is applied.

### 3.5. Independent Test-Set Validation of Freshness Level in Mini-Chinese Cabbage

An external dataset with an additional 135 mini-Chinese cabbages was created using FT-NIR spectroscopy. Independent tests of freshness level were carried out based on the SVC models presented in Section 3.4 in order to verify the robustness of the model. The final results, dividing freshness levels into three categories, are summarized in Table 6. An accuracy of 82.2% was obtained for determinations of freshness levels in independent datasets. A decrease of 6.6% of the accuracy regarding freshness levels was found during external validation, indicating that the SVC model offers substantial potential for the evaluation of freshness of mini-Chinese cabbage.

## 4. Conclusions

This study described the use Fourier transform-near infrared (FT-NIR) spectroscopy with reflectance pattern to quantify the quality attributes of mini-Chinese cabbage. The results indicated that the proposed prediction model is suitable for evaluations of weight loss rate, surface color, and Vc, but not for firmness quantification, in mini-Chinese cabbage stored in nanopackaging. As such, we demonstrated that FT-NIR has the potential to be a useful tool for the evaluation of freshness levels in mini-Chinese cabbage subject to nanopacking. The consistent accuracy obtained by internal and external validation illustrated that the model is effective and robust. Further validation using a larger number of mini-Chinese cabbages from different batches is required.

## Figures and Tables

**Table 1:** Classification of freshness levels of mini-Chinese cabbage.

Freshness Levels	Description
Level 1	Surface without visible defects, smells fresh. Quality attributes including weight loss rate < 30%, L* > 71 and Vc content > 59 mg/100 g.
Level 2	Surface with visible defect points or peculiar smells. Quality attributes including 30% ≤ weight loss rate < 50%, 68 < L* ≤ 71 and 47 < Vc content ≤ 59 mg/100 g.
Level 3	Surface with visible defect areas and unpleasant smell. Quality attributes including weight loss rate ≥ 51%, L* ≤ 68 and Vc content ≤ 47 mg/100 g.

Note: if one item did not meet the requirement, the freshness level was decreased.

**Table 2:** Performance of the regression models for weight loss ratio, firmness and vitamin C content of individual mini-Chinese cabbages.

Quality Attributes	Pretreatment	Model	Calibration		Cross-Validation		Prediction		
			*R_C_* ^2^	RMSEC	*R_CV_* ^2^	RMSECV	*R_p_* ^2^	RMSEP	RPD
Weight loss rate	SNV	PLSR	0.95	1.334	0.92	1.339	0.96	1.332	3.612
	1-st		0.90	1.340	0.86	1.573	0.87	1.360	2.191
	2-nd		0.82	1.654	0.80	1.732	0.80	1.564	3.212
	MSC		0.88	1.365	0.85	1.537	0.88	1.354	2.435
	Autoscale		0.86	1.573	0.81	1.691	0.84	1.476	2.830
	SNV	SVR	0.83	1.643	0.80	1.733	0.79	1.590	2.700
	1-st		0.89	1.378	0.87	1.520	0.87	1.359	2.191
	2-nd		0.85	1.587	0.81	1.720	0.83	1.489	2.795
	MSC		0.87	1.520	0.82	1.647	0.85	1.435	2.546
	Autoscale		0.87	1.489	0.82	1.649	0.85	1.461	2.544
Firmness	SNV	PLSR	0.58	2.542	0.50	2.714	0.57	2.606	1.437
	1-st		0.60	2.403	0.51	2.704	0.58	2.578	2.066
	2-nd		0.57	2.604	0.49	2.821	0.44	2.706	1.128
	MSC		0.51	2.704	0.44	2.781	0.51	2.695	2.042
	Autoscale		0.45	2.775	0.40	3.305	0.40	2.789	1.195
	SNV	SVR	0.60	2.463	0.51	2.812	0.57	2.598	1.608
	1-st		0.55	2.671	0.48	2.901	0.50	2.701	2.159
	2-nd		0.58	2.534	0.47	2.953	0.49	2.735	2.124
	MSC		0.56	2.638	0.41	3.217	0.48	2.780	2.091
	Autoscale		0.60	2.479	0.55	2.671	0.60	2.453	2.205
Vitamin C	SNV	PLSR	0.90	3.213	0.85	3.474	0.86	3.43	2.727
	1-st		0.91	3.231	0.84	3.481	0.89	3.25	2.883
	2-nd		0.81	3.500	0.75	4.002	0.80	3.46	2.015
	MSC		0.90	3.113	0.85	3.474	0.95	3.19	2.681
	Autoscale		0.87	3.453	0.74	4.110	0.78	3.48	2.113
	SNV	SVR	0.82	3.496	0.74	4.024	0.79	3.47	2.238
	1-st		0.86	3.467	0.81	3.500	0.87	3.24	2.513
	2-nd		0.88	3.405	0.80	3.557	0.87	3.25	2.512
	MSC		0.89	3.436	0.81	3.501	0.85	3.46	2.442
	Autoscale		0.90	3.298	0.80	3.557	0.82	3.31	2.379

Note: Underline indicates the best prediction performance.

**Table 3:** Performance of the regression models for surface color of individual mini-Chinese cabbages.

Quality Attributes	Pretreatment	Model	Calibration		Cross-Validation		Prediction		
			*R_C_* ^2^	RMSEC	*R_CV_* ^2^	RMSECV	*R_p_* ^2^	RMSEP	RPD
L*	SNV	PLSR	0.72	2.571	0.68	2.823	0.70	2.324	2.458
	1-st		0.69	2.803	0.62	3.074	0.67	2.415	2.258
	2-nd		0.74	2.472	0.67	2.854	0.71	2.183	2.015
	MSC		0.77	2.372	0.68	2.823	0.72	2.051	2.453
	Autoscale		0.65	2.762	0.60	3.227	0.60	2.903	2.469
	SNV	SVR	0.74	2.586	0.64	2.974	0.65	2.372	1.541
	1-st		0.73	2.594	0.65	2.914	0.74	2.134	2.445
	2-nd		0.68	2.908	0.61	3.146	0.60	2.961	2.098
	MSC		0.84	2.051	0.74	2.586	0.82	2.013	3.069
	Autoscale		0.80	2.162	0.71	2.584	0.75	2.122	2.483
a*	SNV	PLSR	0.75	1.241	0.68	1.586	0.72	1.309	2.088
	1-st		0.70	1.443	0.62	1.733	0.68	1.528	1.781
	2-nd		0.68	1.587	0.61	1.748	0.64	1.691	1.332
	MSC		0.71	1.401	0.62	1.733	0.68	1.528	1.781
	Autoscale		0.74	1.287	0.67	1.593	0.69	1.501	1.855
	SNV	SVR	0.77	1.032	0.71	1.403	0.73	1.288	2.145
	1-st		0.72	1.317	0.68	1.557	0.70	1.402	1.987
	2-nd		0.67	1.594	0.60	1.756	0.65	1.625	1.501
	MSC		0.72	1.317	0.67	1.594	0.70	1.402	1.987
	Autoscale		0.75	1.243	0.68	1.557	0.71	1.388	2.051
b*	SNV	PLSR	0.80	1.211	0.71	1.302	0.78	1.278	1.943
	1-st		0.85	1.204	0.72	1.283	0.85	1.264	2.432
	2-nd		0.79	1.236	0.72	1.289	0.72	1.323	1.893
	MSC		0.81	1.218	0.73	1.301	0.73	1.312	1.897
	Autoscale		0.68	1.324	0.61	1.374	0.67	1.421	1.457
	SNV	SVR	0.73	1.278	0.61	1.374	0.70	1.376	1.541
	1-st		0.78	1.245	0.62	1.370	0.73	1.356	1.896
	2-nd		0.78	1.234	0.62	1.370	0.75	1.321	1.913
	MSC		0.80	1.216	0.72	1.289	0.74	1.310	1.906
	Autoscale		0.77	1.270	0.70	1.311	0.71	1.368	1.632

Note: Underline indicates the best prediction performance.

**Table 4:** Accuracy of classification models of freshness levels in mini-Chinese cabbage by PLS-DA.

Models		Freshness Levels			Accuracy/%
Calibration		Level 1	Level 2	Level 3	
	Level 1	40	1	4	88.8
	Level 2	8	32	5	71.1
	Level 3	2	3	39	86.6
	Total accuracy/%	82.1			
Prediction	Level 1	13	1	1	86.6
	Level 2	1	11	3	73.3
	Level 3	0	3	12	80.0
	Total accuracy/%	79.9			

**Table 5:** Accuracy of classification models of freshness levels in mini-Chinese cabbage by SVC.

Models		Freshness Levels			Accuracy/%
Calibration		Level 1	Level 2	Level 3	
	Level 1	43	1	1	95.5
	Level 2	4	38	3	84.4
	Level 3	1	4	40	88.8
	Total accuracy/%	89.6			
Prediction	Level 1	14	1	0	93.3
	Level 2	0	13	2	86.6
	Level 3	0	2	13	86.6
	Total accuracy/%	88.8			

**Table 6:** External validation for freshness levels in mini-Chinese cabbage by SVC.

Models		Freshness Levels			Accuracy/%
SVC		Level 1	Level 2	Level 3	
	Level 1	38	6	1	84.4
	Level 2	7	34	4	75.6
	Level 3	0	6	39	86.7
	Total accuracy/%	82.2%			

## Data Availability

Not applicable.
